# Comparison of the efficacy and safety between rivaroxaban and dabigatran in the treatment of acute portal vein thrombosis in cirrhosis

**DOI:** 10.1186/s12876-023-02960-8

**Published:** 2023-09-25

**Authors:** Haonan Zhou, Mingdong Wu, Shixiong Yu, Han Xia, Wu Yu, Kai Huang, Yikuan Chen

**Affiliations:** 1 Department of Vascular Surgery, The Second Affiliated Hospital of Chongqing Medical University, #76 Linjiang Road, Yuzhong District, Chongqing, 400010 People’s Republic of China; 2 Department of General Surgery, The First Affiliated Hospital of Chongqing Medical and Pharmaceutical College, Chongqing, 400060 People’s Republic of China; 3 Department of Cardiothoracic Surgery, Second Clinical Hospital, Army Medical University, Chongqing, 400000 People’s Republic of China

**Keywords:** Acute portal vein thrombosis, Rivaroxaban, Dabigatran, Cirrhosis

## Abstract

**Background:**

New oral anticoagulants (NOACs) have been becoming prevalent in recent years and are increasingly used in the treatment of port vein thrombosis. The difference of the efficacy and safety between rivaroxaban and dabigatran remains unclear in the treatment of cirrhotic patients with acute portal vein thrombosis (PVT).

**Methods:**

This retrospective study included all consecutive cirrhotic patients with acute portal vein thrombosis in our institute from January 2020 to December 2021. The patients received oral anticoagulation with rivaroxaban or dabigatran. The demographic, clinical, and imaging data of patients were collected. The diagnosis of acute PVT was confirmed by imaging examinations. The severity of liver cirrhosis was assessed using Child–Pugh score and Model for End-Stage Liver Disease (MELD) score. Outcomes included recanalization (complete, partial, and persistent occlusion), liver function, bleedings, and survival. The log-rank test was used to compare Kaplan–Meier distributions of time-to-event outcomes. The Cox proportional hazards model was used to calculate hazard ratios (HRs) with 95% confidence intervals (CIs).

**Results:**

A total of 94 patients were included, 52 patients (55%) received rivaroxaban and 42 (45%) with dabigatran. The complete and partial recanalization of PVT was observed in 41 patients. There was no significant difference in complete recanalization, partial recanalization, and persistent occlusion between the two groups. With multivariate analysis, D-dimer (HR 1.165, 95% CI 1.036–1.311, *p* = 0.011) was independent predictors of complete recanalization. The Child–Pugh score (*p* = 0.001) was significantly improved in both two groups after anticoagulation, respectively. However, there was no difference between the two groups. The probability of survival was 94%, 95% in the rivaroxaban and dabigatran groups (log-rank *p* = 0.830). Major bleedings were reported in 3 patients (6%) in rivaroxaban group and 1 patient (2%) in dabigatran group (*p* = 0.646). Six patients (12%) in rivaroxaban group experienced minor bleeding, and five (12%) from dabigatran group (*p* = 0.691).

**Conclusions:**

The efficacy and safety were comparable between rivaroxaban and dabigatran in the treatment of cirrhotic patients with acute portal vein thrombosis. And D-dimer can contribute to the prediction of PVT recanalization in cirrhotic patients.

## Introduction

Acute Portal vein thrombosis (PVT) is a potential thrombotic complication in cirrhotic patients with a prevalence ranging between 0.6% and 26% [1–3]. And it has been correlated with gastrointestinal bleeding, the onset and development of ascites, or hepatic encephalopathy [4].

Anticoagulation is usually recommended as the first-line therapy for PVT. The benefits of anticoagulation are approved in cirrhotic patients, including a higher recanalization rate, a lower rate of thrombus progression, a reduced incidence of hepatic decompensation, and increased longevity [5]. Although the concern of an increased bleeding risk prevents some cirrhotic patients with PVT from receiving anticoagulant therapy, most studies indicated that anticoagulation appears to be rather safe [6–9]. The practice guidelines and consensus statements recommend low-molecular-weight heparin (LMWH) or vitamin K antagonists (VKAs) for the treatment of acute PVT [10, 11]. When compared to these traditional anticoagulants, the new oral anticoagulants (NOACs) rivaroxaban and dabigatran are increasingly used in clinical practice because they do not require routine laboratory monitoring and has fewer food-drug interactions [12]. Additionally, several recent reports have suggested that NOACs are quality-equivalent or even superior to LMWH or VKAs in the prevention and treatment of venous thromboembolism (VTE) [13–15].

The NOACs rivaroxaban and dabigatran have demonstrated to have comparable efficacy in the prophylaxis and treatment of VTE [16–18]. Liver function in patients with cirrhosis is impaired to varying degrees, and the occurrence of PVT may lead to worsening liver function. Meanwhile 65% of rivaroxaban and only 20% of dabigatran are eliminated by the liver [19, 20]. Therefore, we suspect that there may be differences in the anticoagulant effect and bleeding risk between the two drugs in patients with cirrhosis. In this retrospective study, we aim to compare rivaroxaban and dabigatran with a focus on the efficacy and safety for the treatment of PVT in cirrhotic patients.

## Method

### Patient enrollment

Patients diagnosed with acute PVT were identified by searching the electronic medical records at our large urban tertiary care center from January 2020 to December 2021. This study was approved by our Institutional Ethics Committee. Inclusion criteria were: (1) acute PVT: developed symptoms < 60 days [21]; (2) age ≥ 18 years old; (3) the diagnosis of PVT and cirrhosis was confirmed by contrast-enhanced computed tomography (CT), magnetic resonance imaging (MRI) or Doppler ultrasound; (4) liver function: Child–Pugh grade A or grade B. Exclusion criteria: (1) chronic PVT: the presence of cavernous transformation of the portal vein on images and/or symptoms more than 60 days [21]; (2) contraindications for anticoagulation: active bleeding, renal insufficiency (creatinine clearance < 50 ml/min); (3) low-dose anticoagulation: less than standard (dabigatran 75 mg or rivaroxaban 10 mg or 15 mg); (4) hepatocellular carcinoma or tumor thrombosis; (5) isolated thrombosis of superior mesenteric vein or splenic vein without portal vein involvement; (6) interventional therapy by thrombolysis or transjugular intrahepatic portosystemic shunt (TIPS) and bowel resection due to necrosis of the intestine; (7) absence of radiology imaging for PVT diagnosis; (8) absence of subsequent follow-up imaging 3 or more months after diagnosis; (9) Child–Pugh grade C liver function; (10) platelet count < 20 × ${10}^{9}$/L. The NOACs are not recommended in severe cirrhosis with Child–Pugh grade C due to extremely high bleeding risk [22]. In addition, we excluded patients with renal function < 50 ml/min to avoid the bias on dabigatran because dabigatran is excreted predominantly by the kidneys and its dosing is dependent on kidney function and creatinine clearance [23]. All registered patients were divided into two groups (rivaroxaban group and dabigatran group) according to the type of anticoagulants.

The patients’ gender, age, etiology of cirrhosis, follow-up period, comorbidities, laboratory tests (including D-dimers, liver function tests, blood platelet), and classification of PVT according to the Yerdel classification were recorded. Thrombocytopenia was defined as platelet count < 100 x ${10}^{9}$/L [24]. The Yerdel grading system includes: grade 1, < 50% occlusion of main portal vein with no or minimal obstruction of superior mesenteric vein (SMV); grade 2, > 50% obstruction of main portal vein; grade 3, complete obstruction of main portal vein and proximal SMV; grade 4, complete obstruction of the portal vein and SMV [25]. All patient underwent endoscopic screening and primary prophylaxis for variceal bleeding according to the Baveno VII consensus guidelines [26]. Esophageal varices were classified as small (F1), medium (F2), and large (F3) based on presence and size. To assess severity of cirrhosis, Child–Pugh score and Model for End-Stage Liver Disease (MELD) score were calculated at baseline and the end of the follow-up period. The follow-up data was collected by phone call.

All enrolled patients were treated with low molecular weight heparin (4000 anti-XA IU/0.4 ml) within 1–2 days as a bridging therapy after admittance. During treatment, the full recommended doses of rivaroxaban and dabigatran were administered, particularly 15 mg bid on 1–20 days then 20 mg qd of rivaroxaban, 150 mg or 110 mg bid of dabigatran.

The primary outcome was recanalization (complete, partial, and persistent occlusion). The MRI and CT imaging were performed to assess recanalization of the acute portal vein thrombosis. Secondary outcomes included liver function, survival and bleedings. Complete recanalization was defined as the disappearance of prior PVT. Partial recanalization referred to a more than 50% reduction of the thrombus, without the thrombus extending to other veins [27]. Persistent occlusion was defined as that the thrombus maintained the same dimension and failed to reach recanalization. Major bleeding was defined according to the ISTH as clinically overt bleeding associated with a fall in hemoglobin by >  = 20 g/L, transfusion of >  = 2 U packed red blood cells or whole blood, retroperitoneal or intracranial bleeding, or fatal bleeding [28]. Minor bleeding events were defined as all other clinically significant bleeding events without need for transfusion or intervention [28].

### Statistical analysis

Continuous variables are described by mean ± standard deviation (SD) or medians with interquartile range (IQR) according to data distribution. Categorical variables were summarized as frequencies. The continuous variables were compared with Student's t-test (normally distributed) or Mann–Whitney test (non-normally distributed), while categorical variables were compared by chi2 test. Univariate analysis and multivariate analysis were performed to calculate hazard ratios (HRs) and corresponding 95% confidence intervals (CIs) by Cox proportional hazards model, and to find significant variables associated with recanalization. Backwards selection with 0.05 significance level was used to select variables for inclusion in the final multivariable models. Survival curves were estimated using the Kaplan–Meier method and compared with the log-rank test. All *P*-values were two-tailed and a *P* value of < 0.05 was considered significant. Statistical analysis was performed using SPSS 26.0 (IBM, Armonk, NY, USA).

## Result

### Study population

**Fig. 1:**
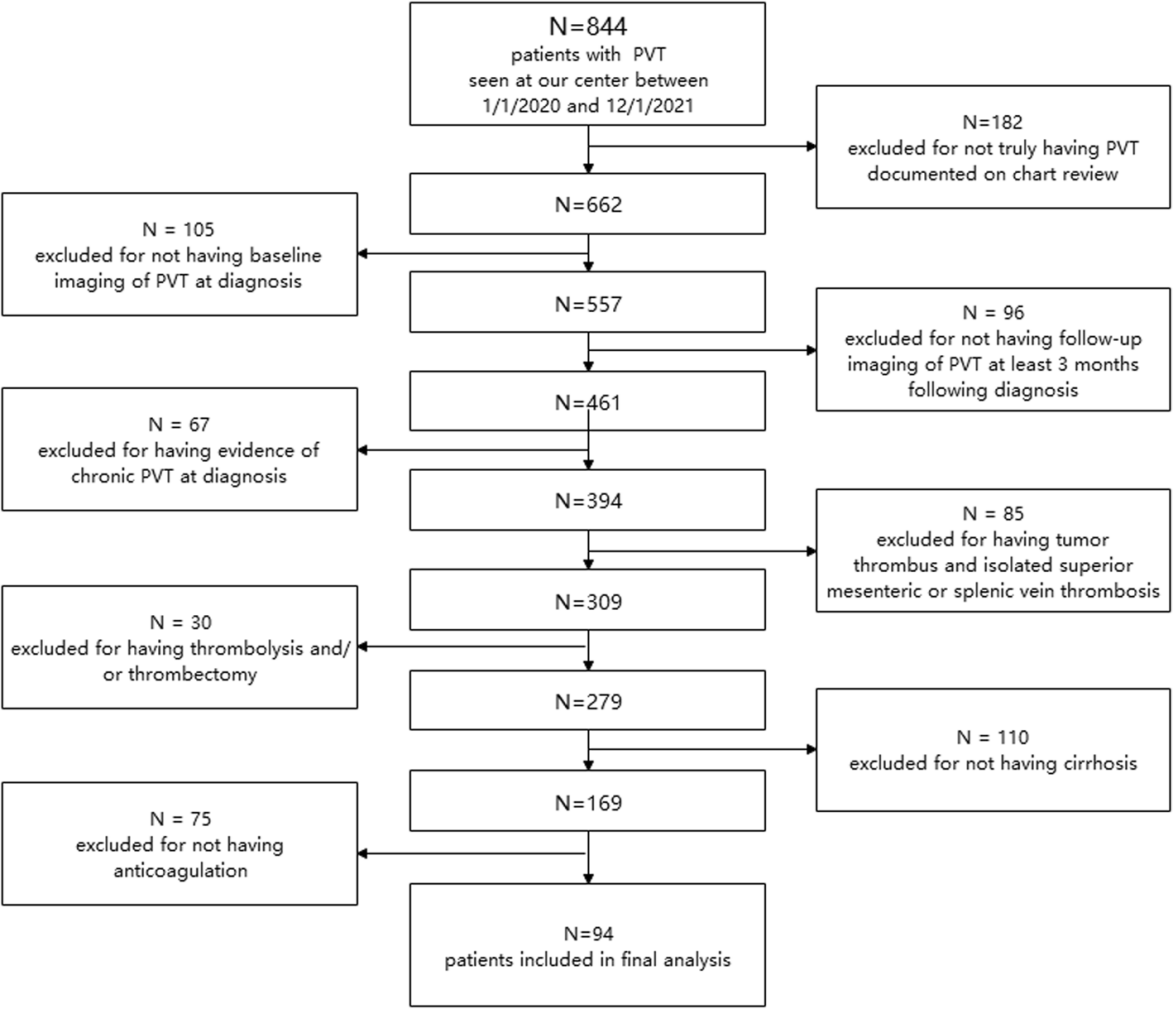
Flow chart of exclusion process of study population

A total of 844 patients with PVT were screened and 94 patients were included in the study. Reasons for exclusion are shown in the CONSORT diagram (Fig. 1). The imaging diagnosis of thrombosis included Doppler ultrasound (*n* = 2), CT (*n* = 88) and MRI (*n* = 4). And the follow-up imaging consisted of ultrasound (*n* = 11), CT (*n* = 80), or MRI (3). Mean Child Pugh score and MELD score were 7.02 ± 1.14 and 8.70 ± 3.95 respectively. Thrombocytopenia was observed in 36% of patients (34/94). Approximately 22% of patients (21/94) had complete occlusive portal vein thrombosis. Superior mesenteric and splenic vein were involved in 24 (25%) cases. Twenty-seven patients (27/94, 29%) were on beta-blocker therapy and only seven patients (7/94, 7%) underwent variceal band ligation as prophylaxis of variceal bleeding. About four patients (4/94, 4%) with history of previous esophageal varices bleeding underwent endoscopic sclerotherapy. In terms of the severity of gastroesophageal varices, F3, F2 and F1 were observed in 1, 10 and 30 patients in the rivaroxaban group, and 1, 4 and 30 patients in the dabigatran group.

There were 52 patients (55%) in rivaroxaban group and 42 patients (45%) in dabigatran group. During follow-up, all patients were remained in their initial anticoagulation group for analysis. Demographics and baseline characteristics were compared between two groups (Table 1). All patients received at least 3 months of anticoagulation. The median duration of oral anticoagulant treatment was 15 months (IQR 6–24 months) in the rivaroxaban group and 18 months (IQR 8–24 months) in the dabigatran group (*p* = 0.643). And the median follow-up duration of all patients was 36 months (IQR 31–36 months) and 36 months (IQR 32–36 months) in two groups, respectively (*p* = 0.686).

**Table 1:** Baseline characteristics of patients treated with Rivaroxaban or Dabigatran

Variable	Rivaroxaban *n* = 52	Dabigatran *n* = 42	*P* Value
Age (y), mean (SD)	55 (14)	55 (13)	0.828
Sex (male), n (%)	38 (73)	23 (55)	0.111
Esophageal varices F1/F2/F3, n (%)	30/9/1 (32/10/1)	28/4/1 (30/4/1)	0.849
Beta-blocker therapy, n (%)	15 (29)	12 (29)	0.977
Etiology of cirrhosis, n (%)			0.314
Alcohol	14 (27)	6 (14)	
HBV	28 (54)	21 (50)	
HCV	1 (2)	1 (2)	
Autoimmune	3 (6)	8 (19)	
NASH	2 (4)	1 (2)	
Other	4 (8)	5 (12)	
Child–Pugh score, mean (SD)	7.1 (1.1)	6.9 (1.2)	0.284
MELD score, mean (SD)	9.0 (7.0–10.5)	8.4 (7.4–10.7)	0.834
D-dimer (ug/mL),(IQR)	1.7 (0.8–3.2)	2.8 (0.9–5.1)	0.127
INR − median (IQR)	1.2 (1.1–1.4)	1.2 (1.1–1.4)	0.083
Total bilirubin (umol/L), mean (SD)	23 (23)	22 (20)	0.667
Albumin (g/L), mean (SD)	29 (5)	30 (5)	0.468
Creatinine (mmol/L), mean (SD)	67.2 (18.9)	65 (17.6)	0.56
GFR (mL/ min), mean (SD)	104 (23)	101 (21)	0.396
Platelet (x10^9/L), (IQR)	126 (63–203)	179 (66–258)	0.115
Duration of LMWH bridging therapy	1 (1–2)	1 (1–2)	0.615
Yerdel classification, n (%)			0.637
Grade I	15 (19)	11 (26)	
Grade II	11 (21)	11 (26)	
Grade III	10 (19)	12 (27)	
Grade IV	16 (31)	8 (11)	
Complete occlusive thrombus, n (%)	12 (23)	9 (21)	0.849

*HBV* Hepatitis B virus, *HCV* Hepatitis C virus, *NASH* Nonalcoholic steatohepatitis, *MELD score* Model for End-Stage Liver Disease, *INR* International normalized ratio, *GFR* Glomerular filtration rate

### PVT recanalization

**Fig. 2:**
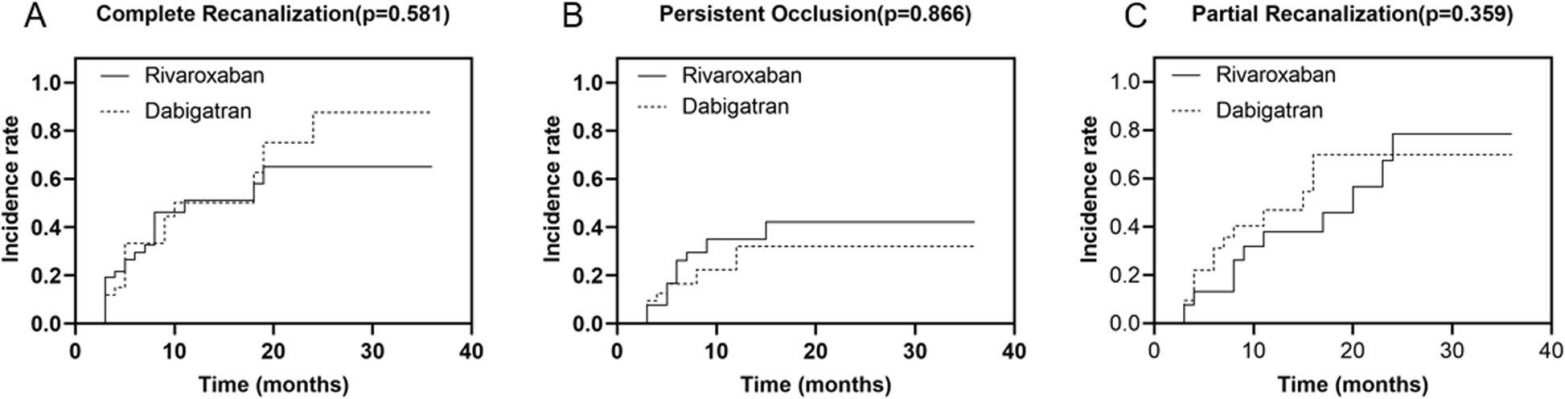
Kaplan–Meier curves for rivaroxaban and dabigatran groups. Complete recanalization (**A**), partial recanalization (**B**) and persistent occlusion (**C**) of PVT

The Kaplan–Meier curves depicted the likelihood of recanalization over time among patients who received rivaroxaban or dabigatran (Fig. 2).

The complete and partial recanalization rate was 75% (39/52) and 79% (33/42) in the rivaroxaban and dabigatran groups, respectively. The complete recanalization of the portal vein thrombosis was achieved in 24 patients in the rivaroxaban group and 17 patients in the dabigatran group (Fig. 2A). The rivaroxaban group had a higher rate of complete recanalization but not significantly different relative to the dabigatran group (46% vs. 40%, log-rank *p* = 0.581). In the univariate analysis, esophageal varices (HR 0.501, 95% CI 0.258–0.973, *p* = 0.041) and D-dimer (HR 1.188, 95% CI 1.058–1.334, *p* = 0.004) were predictors associated with the complete recanalization of PVT. In the multivariate analysis, only D-dimer (HR 1.165, 95% CI 1.036–1.311, *p* = 0.011) was independent predictor for the complete recanalization (Table 2).

**Table 2:** Univariate and multivariate analysis to determine predictive factors for complete recanalization

Variables	HR	95% CI	*P* value	HR	95% CI	*P* value
Male	0.918	0.478–1.764	0.798			
Age (years)	1.015	0.992–1.039	0.197			
Esophageal varices	0.501	0.258–0.973	0.041	1.730	0.873–3.429	0.116
NOACs	1.084	0.578–2.031	0.801			
Child–Pugh score	0.930	0.703–1.232	0.614			
MELD score	0.967	0.891–1.05	0.426			
Total bilirubin (umol/L)	0.996	0.978–1.014	0.667			
Albumin (g/L)	1.033	0.970–1.099	0.310			
GFR (mL/ min)	0.991	0.977–1.006	0.230			
INR	0.328	0.068–1.584	0.165			
D-dimer (ug/mL)	1.188	1.058–1.334	0.004	1.165	1.036–1.311	0.011
Platelet (x10^9/L)	1.001	0.999–1.004	0.322			
Yerdel classification	1.006	0.777–1.302	0.962			
Complete occlusive thrombus	1.022	0.504–2.074	0.952			

*HR* Hazard ratio, *CI* Confidence interval, *NOACs* New oral anticoagulants, *MELD score* Model for End-Stage Liver Disease, *INR* International normalized ratio, *GFR* Glomerular filtration rate

Among the 94 patients, 21 patients had complete occlusive PVT (rivaroxaban group: 12/52, 23%; dabigatran group: 9/42, 21%). The rivaroxaban group was more likely to have occlusive PVT than dabigatran group (23% vs. 21%). However, there was no statistically difference between the groups (*p* = 0.849). In the two groups, 7/12 (58%) and 4/9 (44%) patients respectively achieved complete recanalization without statistically significant difference (log-rank *p* = 0.827).

Fifteen patients in the rivaroxaban group and sixteen patients in the dabigatran group attained partial recanalization (Fig. 2B). There was no statistically significant difference between the two groups (29% vs. 38%, log-rank *p* = 0.359). No variables were found to be significantly associated with the rate of partial recanalization within the univariate cox analysis. While in the multivariate Cox proportional hazards model, D-dimer (HR 1.195, 95% CI 1.063–1.343, *p* = 0.003) was significantly associated with partial recanalization of PVT.

In addition, 13 patients in the rivaroxaban group and 9 patients in the dabigatran group experienced persistent occlusion (Fig. 2C). But the rate of persistent occlusion was not different in the two groups (21% vs. 19%, log-rank *p* = 0.866). In the univariate analysis, Child–Pugh score (HR 1.782, 95% CI 1.200–2.645, *p* = 0.004), MELD score (HR 1.108, 95% CI 1.010–1.215, *p* = 0.030), total bilirubin (HR 1.020, 95% CI 1.008–1.032, *p* = 0.001), and INR (HR 3.517, 95% CI 1.337–9.250, *p* = 0.011) were significantly associated with persistent occlusion. However, none of the variables were independent predictors in multivariate Cox regression analysis.

After anticoagulation therapy, the values of D-dimer were significantly decreased in the rivaroxaban and dabigatran groups (2.50 mg/l [0.77–3.24] vs. 1.49 mg/l [0.35–1.92], *p* = 0.015; 3.25 mg/l [0.93–5.12] vs. 1.42 mg/l [0.49–1.84], *p* = 0.001). But no statistically significant difference was found between the two groups (1.01 ± 2.88 mg/l vs. 1.83 ± 3.01 mg/l, *p* = 0.182).

### Scoring systems

**Fig. 3:**
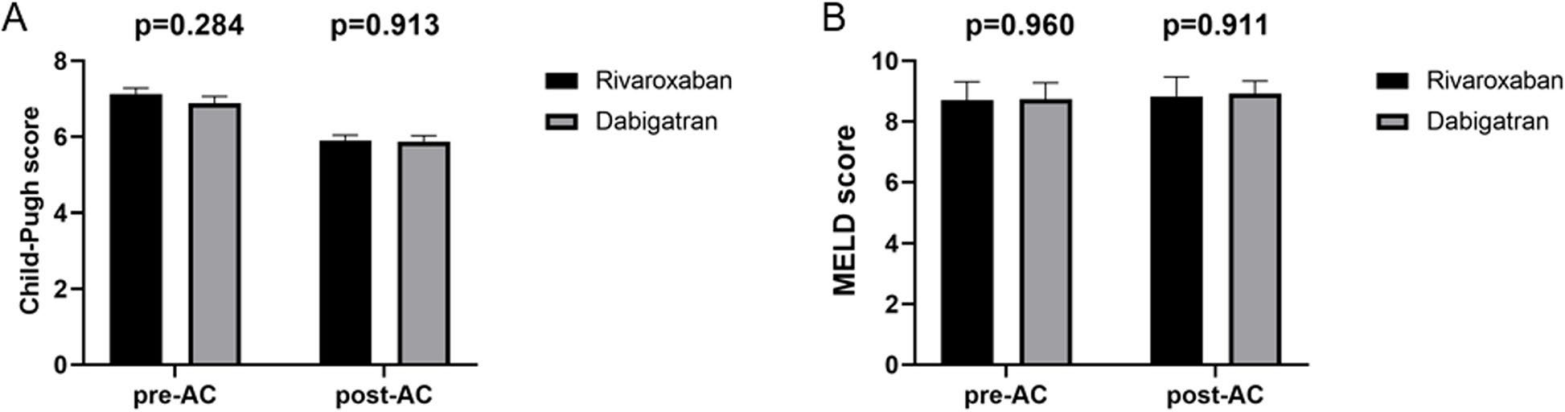
Comparison of Child–Pugh score and MELD score in the Rivaroxaban and Dabigatran groups. MELD score, Model for End-Stage Liver Disease score. AC, Anticoagulation

The mean Child–Pugh score of the rivaroxaban group was 7.13, and 6.88 in the dabigatran group without significant difference (*p* = 0.284). The Child–Pugh score was improved in the rivaroxaban group (7.13 ± 1.09 vs. 5.90 ± 1.05, *p* = 0.001) and dabigatran group (6.88 ± 1.19 vs. 5.88 ± 0.97, *p* = 0.001) after anticoagulation. Comparisons between the two groups (pre- and post-treatment), there was no statistically significant difference between the two groups (Fig. 3A). No significant improvement of MELD score was observed in rivaroxaban group (8.70 ± 4.40 vs. 8.83 ± 4.59, *p* = 0.877) and dabigatran group (8.74 ± 3.47 vs. 8.92 ± 2.73, *p* = 0.792) after anticoagulation. In addition, no statistical differences occurred between the two groups (Fig. 3B).

### Survival and bleeding

**Fig. 4:**
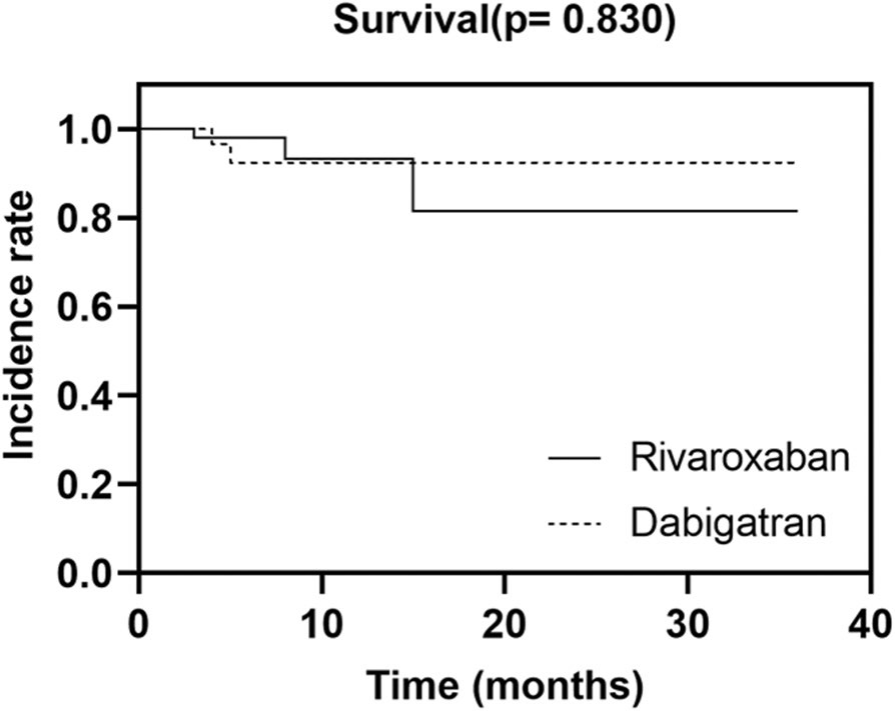
Survival by anticoagulation between the Rivaroxaban and Dabigatran groups

During follow-up, 5 patients died (3 were on rivaroxaban and 2 on dabigatran): two patients died of liver failure, two patients died due to septic shock, and in one patient the cause of death was unknown. As depicted in the survival curve of Fig. 4, the probability of survival did not differ between patients receiving rivaroxaban and dabigatran (94% vs. 95%, log-rank *p* = 0.830). In the univariate analysis, age (HR 1.077, 95% CI 1.006–1.154, *p* = 0.034) and GFR (HR 0.942, 95% CI 0.897–0.990, *p* = 0.017) were associated with the mortality. The small number of deaths precluded multivariate analysis.

Bleeding events was observed in this study with 9 patients (17%) in rivaroxaban group and 6 patients (14%) in dabigatran group (*p* = 0.692). Major bleedings occurred in 3 patients in rivaroxaban group (2 with variceal bleeding and 1 with intracranial hemorrhage) and 1 patient in dabigatran group (variceal bleeding). However, there were no significant differences for major and minor bleeding events between rivaroxaban and dabigatran groups (6% vs. 2%, *p* = 0.646; 12% vs. 12%, *p* = 0.691). Notably, only INR (HR 4.306, 95% CI 1.378–13.456, *p* = 0.012) was associated with bleeding risk.

## Discussion

The use of NOACs in the management of cirrhotic patients with PVT remains contentious. Nowadays, a number of studies demonstrated that NOACs have superiority or noninferiority to VKAs or LMWH in reducing risk of thromboembolic complications with similar or reduced bleeding risk [29–32]. Our results showed that the recanalization rate of PVT in the rivaroxaban group was comparable to the dabigatran group and with no statistically significant difference in bleeding rate.

These two kinds of drugs (rivaroxaban and dabigatran) differ in various ways. Firstly, they have different drug metabolic and elimination pathways. Approximately one-third of rivaroxaban is eliminated unchanged by the kidneys and about two-thirds of the drug being metabolized by the liver, and half is eliminated via the kidneys and another half via the fecal route. While more than 80% of dabigatran is excreted via the renal pathway [33, 34]. Secondly, rivaroxaban, as one of the direct factor Xa inhibitor, reversibly inhibits clot-bound factor Xa and prothrombinase activity without affecting existing thrombin levels to affect both the intrinsic and extrinsic pathways of the coagulation cascade. While dabigatran is capable of rapidly and reversibly bind with both free and clot-bound thrombin, thus preventing the conversion of fibrinogen to fibrin [35, 36]. Notably, a recent study suggested that the in vitro anticoagulant effect of rivaroxaban was decreased in patients with liver cirrhosis and the enhanced effect of dabigatran was proportional to the severity of liver disease [37]. It provides a new idea for future clinical and basic research.

Despite these differences, the clinical efficacy and safety of rivaroxaban and dabigatran appear to be quite similar in this study. Multiple studies have shown that that the efficacy and safety were comparable between them in patients with nonvalvular atrial fibrillation or venous thromboembolism [38–40]. Likewise, we found rivaroxaban and dabigatran showed similar efficacy in the rate of complete recanalization, partial recanalization and persistent occlusion for the treatment of PVT in this study. Only patients in Child–Pugh grade A and B were included in this study, and the effect of Child–Pugh grade C on the metabolism of rivaroxaban and dabigatran was not considered. Besides, the changes in the in vivo anticoagulant effect of rivaroxaban and dabigatran in cirrhotic PVT remains unclear [41]. These may explain that similar efficacy was observed between dabigatran and rivaroxaban in our study. And these results were more likely to motivate further studies to discern the effectiveness of rivaroxaban and dabigatran for treatment of PVT in cirrhotic patients.

In this study, D-dimer was an independent predictor of PVT recanalization including both complete and partial recanalization according to univariate and multivariate analysis. Zhou et al. [27] reported that D-dimer was associated with recanalization of PVT in a randomized controlled trial. Another multi-centric randomized controlled trial by Gao et al. [42] demonstrated that D-dimer < 2.00 ug/mL (*P* = 0.030, OR: 3.600, 95% CI 1.134–11.430) was one of the predictors of PVT recanalization through univariate analysis. Further studies with a larger number of patients are required to confirm it.

Existing studies suggested that the anticoagulation treatment may improve liver function [27, 43]. In this study, the improvements of the Child–Pugh score were quite significant after anticoagulation therapy. However, no significant difference was observed between the two groups. This may be because liver function affected by both drugs at comparable levels in cirrhosis. All patients in this study had relatively good liver function reserves, and the baseline Child–Pugh score averaged 7.02. Therefore, this study has not adequately assessed the effect of anticoagulation in cirrhotic patients with Child–Pugh score C. And future studies included patients with Child–Pugh score C are required to provide more convincing evidence.

On the other hand, an increased risk of bleeding, especially esophageal varices, is concerned in cirrhotic patients who need anticoagulant therapy. However, data that demonstrate therapeutic AC may not increase bleeding risk among those patients are increasingly emerging [2, 44]. In a meta-analysis of real-world studies, 2 studies involving 923 patients showed no significant difference of major bleeding between dabigatran and rivaroxaban (*p* = 0.25) [45]. Note that no significant association was found between platelet count or Child–Pugh Grade and major bleeding. However, some case reports have found that dabigatran is associated with exfoliative esophagitis and esophageal ulcer [46–48]. Toyo et al. displayed that 19 (20.9%) of the 91 patients receiving dabigatran showed esophagitis in a retrospective study [49]. No symptomatic esophagitis was observed among patients taking dabigatran in our study, which can be attributable to the relatively low number of cases and a majority of patients failed to perform routine esophagoscopy within the follow-up period.

Given the retrospective nature of our study, it has some notable limitations. Firstly, some adverse and bleeding events may have been underreported. Besides, physician choice of anticoagulation may have been influenced by unknown and uncontrolled variables. Indeed, it is impossible to adjust for all possible confounding variables in such a study, and this kind of comparison has a great likelihood of selection bias. As a result, the definitive conclusions regarding the relative safety and efficacy across anticoagulants may not be drawn. In addition, some recanalization and other events noted more than a year after initiation of anticoagulation may be likely occurred much earlier because some patients receiving follow-up scans at frequent and regular interval, and others waiting long periods of time before their first follow-up imaging. Hopefully, the findings presented may encourage the performance of a randomized controlled trial. Strengths of this study include strict inclusion criteria, long periods of follow-up, and robust statistical analysis.

In conclusion, both rivaroxaban and dabigatran appeared effective and can be advised to treat acute portal vein thrombosis for cirrhotic patients. And the two groups achieved a higher rate of complete recanalization without a significant increase in bleeding. In addition, D-dimer can be served as a potential predictor of PVT recanalization in cirrhotic patients.

## Data Availability

The authors confirm that the data supporting the findings of this study are available within its supplementary materials.
